# Word length vs. lexical factors: Re-examining what causes the word-length effect in serial recognition

**DOI:** 10.3758/s13421-025-01762-5

**Published:** 2025-09-08

**Authors:** Dominic Guitard, Ian Neath, Aimée M. Surprenant

**Affiliations:** 1 School of Psychology, Cardiff University, Cardiff, UK; 2 Department of Psychology, Virginia Tech, Blacksburg, VA 24061 USA

**Keywords:** Working memory, Serial recognition, Word length effect, Orthographic neighborhood, Phonological neighborhood

## Abstract

The word-length effect refers to the finding that memory on many short-term/working memory tasks is better for words with fewer syllables than words with more syllables. The standard account attributes this result to a combination of decay offset by rehearsal: More short words can be rehearsed because they take less time to articulate. However, most studies have confounded length with lexical and other long-term memory factors that covary with length. In this paper, we reexamine word-length effects in serial recognition. Experiment 1 replicated previous findings of a word-length effect when short and long words also differed on numerous other dimensions. Experiment 2 found that when the short and long words were more fully equated, including being equated for orthographic and phonological neighborhood size, the word-length effect disappeared. Experiment 3 confirmed that memory was better for words with more orthographic and phonological neighbors than words with fewer neighbors, showing serial recognition is sensitive to at least some lexical/long-term memory factors. The results provide more evidence against the standard account of the word-length effect and instead are consistent with a growing body of work which shows that lexical and other long-term memory factors affect performance in short-term/working memory tasks.

**Supplementary Information:**

The online version contains supplementary material available at 10.3758/s13421-025-01762-5.

In 1935, Calhoon reported a study which found that when people recall a list of words in order, the proportion correct “is in an inverse relation to their syllabic length” (p. 620), a result which came to be known as the word-length effect. Although serial recall is the most common test, the word-length effect has been observed in many different tasks including free recall (Watkins, 1972), probe recall (Avons et al., 1994), serial reconstruction of order (Tolan & Tehan, 2005), complex span (LaPointe & Engle, 1990), and serial recognition (Baddeley et al., 2002). In this paper, we reexamine the latter result and assess whether it is the length of the words or lexical/long-term memory factors that typically covary with length that is driving the effect.

## Word length and the standard model

The word-length effect was central to the development of working memory (Baddeley, 1986) and has been called the “best remaining solid evidence” (Cowan, 1995, p. 42) for theoretical accounts that posit a temporary memory store in which items decay over time. These various accounts offer the same explanation for the word-length effect and collectively they have been referred to as the standard model (Nairne, 2002). According to the standard model, items in working memory decay over time, and once they have decayed sufficiently they can no longer be recalled. Decay can be offset by articulatory rehearsal. Whereas the decay rate is assumed to be constant for all items, the rehearsal rate differs because it is assumed to be proportional to the articulation rate—the amount of time needed to say the words. Because of this difference in articulation rate, more short words can be rehearsed in a given amount of time than long words, and therefore short words, on average, will have decayed less than long words. Much of this work uses immediate serial recall but as noted previously, the word-length effect has been reported in many different paradigms with the same explanation.

There are many variations of the standard model. For example, Baddeley’s (1986) version fractionates working memory into multiple components, of which the phonological loop is used for storing and processing verbal information. The decay occurs in the phonological store, and rehearsing the decaying items is done by the articulatory control process. In contrast, Cowan’s (1999) embedded processes model views working memory as activated long-term memory. In this view, it is the activation that decays and rehearsal resets the activation level. What all the versions have in common is the assumption of decay offset by rehearsal (see Miyake & Shah, 1999, for detailed presentations of a number of different versions of the standard model as well as reviews of studies that support predictions of those models) and that is why the word-length effect has played such a prominent role in theory development.

Despite the success and influence of these accounts, the core of the standard model—decay offset by rehearsal—has been called into question. Because the standard model posits that articulation rate is the key factor, it has to predict that words that take less time to articulate, such as *wiggle*, will be better remembered than words that take more time to articulate, such as *voodoo*, despite the fact that the words are equated on other measures of length (i.e., number of phonemes, letters, and syllables)*.* Baddeley et al. (1975, Exp. 3) created such a set of stimuli and confirmed this prediction. This result is known as the time-based word-length effect because length is defined by pronunciation time. The problem is that the stimulus set used by Baddeley et al. is the only one that produces a time-based word length. For example, Neath et al. (2003) reported four experiments that were identical except for the stimuli. Experiment 1 replicated the time-based word-length effect using the Baddeley et al. stimuli. However, Experiment 2 used stimuli created by Caplan et al. (1992) and replicated their null result; Experiment 3 used stimuli created by Lovatt et al. (2002) and replicated their null result; and Experiment 4 used a new set of stimuli that also failed to produce a time-based word-length effect. The three sets of stimuli that failed to produce a time-based word-length effect all differed significantly in articulation time. There is apparently something unique about the Baddeley et al. stimuli, which consistently produce an advantage for words that take less time to say, compared with all other pools tested, which consistently show no such advantage. The problem for the standard model is that if there is no time-based word-length effect, then any explanation that depends on decay offset by rehearsal is called into question. Not only is the explanation for the word-length effect called into question, but these null results are problematic for the core of the standard model itself.

## Other accounts of the word-length effect

If time to articulate a word is not the cause of the word-length effect, then what is? There have been a number of alternative explanations. One possibility is that output time, rather than pronunciation time per se, drives the effect (e.g., Dosher & Ma, 1998). Accounts based on this idea note that it takes longer to write or say or type a long word than a short word. The problem with this explanation is that many different models predict worse memory if output takes longer and therefore observing such a result is not diagnostic. For example, the standard model posits that items decay over time and this decay continues during recall. If recall takes longer, there will be more decay and thus worse performance. However, the same prediction is made by SIMPLE (Brown et al., 2007) but for a very different reason. SIMPLE is a relative distinctiveness model and assumes that people represent items on a log-transformed dimension that can vary depending on the situation. When items are represented on a temporal dimension and the presentation rate is held constant, SIMPLE predicts worse performance as output time increases. The reason is because of the log transformation: The representations of the items become less distinct the longer the time until retrieval. As a third example, Lewandowsky et al. (2004) explained the effect of worse memory after longer output times by invoking interference, which interacts with representations of the items. Thus, observing a difference in memory when output times differ does not differentiate between different theoretical accounts.

The word-length effect has also been explained by retroactive interference. For example, Campoy (2008; see also Campoy, 2011) suggested that as each word is processed, it could potentially interfere with words that have already been presented. This idea has been implemented in a number of models. For example, within the context of the feature model (Nairne, 1990), items are represented as a vector of features. The presentation of a new item causes overwriting of some of the features of the previous item making it less likely that the previous item will be redintegrated. A second example concerns a very different model, the context-activation model (Davelaar et al., 2005), in which items in short-term memory use lateral inhibition to prevent runaway activation. Specifically, presentation of a new item inhibits previously presented items. Whatever the specific mechanism, Campoy suggested that long words cause more retroactive interference than short words by virtue of having more elements (see also Neath & Nairne, 1995).

Other explanations suggest that the word-length effect occurs because of differences on dimensions that covary with length. A well-known example of a dimension that covaries with length is frequency: short words, on average, are of higher frequency than long words (Sears et al., 2006) and because of this, researchers have long equated their short and long words for frequency. However, there are many other dimensions that covary with length, such as orthographic and phonological neighborhood characteristics, and it was not until relatively recently that researchers began to equate their short and long words on these dimensions.

An older definition of an orthographic neighbor is a word that differs from the target by a single letter (Coltheart et al., 1977). For example, orthographic neighbors of *cat* include *bat*, *cot*, and *cap*. A word’s orthographic neighborhood is the set of these neighbors. Similarly, a phonological neighbor is a word that differs from the target by a single phoneme and a word’s phonological neighborhood is the set of these neighbors. These measures covary with length (Sears et al., 2006). More recent definitions allow for the addition or subtraction of letters and phonemes. Yarkoni et al. (2008) proposed a measure called orthographic Levenshtein distance (OLD) which is based on the number of edits required to transform one word to another. Phonological Levenshtein distance (PLD) is the corresponding measure for phonemes. The reason this may be important when studying word-length effects is that short words tend to have more orthographic and phonological neighbors than long words, and memory is better for words with large neighborhoods than those with small neighborhoods (e.g., Allen & Hulme, 2006; Jalbert et al., 2011a, b); Roodenrys et al., 2002).

Another example of a measure that co-varies with length is the frequency of the orthographic and phonological neighbors (Sears et al., 2006). The frequency of orthographic and phonological neighbors of short words tends to be higher than that of the neighbors of long words. The reason that this may be important when studying word-length effects is that words that have the same number of neighbors but have higher frequency neighbors are recalled better than words that have lower frequency neighbors (MacMillan et al., 2024). Very few studies comparing memory for short and long words equate them on neighborhood frequency.

How might neighborhood size and frequency benefit memory? According to Roodenrys’s (2009) redintegration framework, as each word is encountered, it can serve as input to an interactive activation network. Each word will partially activate its orthographic and phonological neighbors which means that words with more neighbors will partially activate more items than words with fewer neighbors. Crucially, the activation from the neighbors feeds back to the original item and because of this, words with more neighbors will receive more activation feedback than words with fewer neighbors. The higher activation levels of words that receive more feedback aids subsequent redintegration. Because short words tend to have more neighbors than long words, short words will receive more feedback activation and therefore will be remembered better than long words. Other lexical dimensions could work in a similar fashion. The prediction, then, is that if two sets of words are not equated on these lexical dimensions, differential activation can cause the words with more neighbors and higher frequency neighbors to be better remembered than the words with fewer neighbors and lower frequency neighbors.

Jalbert et al. (2011a) noted that no previous study looking at word-length effects had equated short and long words for orthographic neighborhood size and as a result, all had confounded length and neighborhood size. They created two sets of stimuli: In both, the short and long words were equated on nine dimensions: Concreteness, familiarity, imageability, acoustic similarity, three different measures of frequency, number of orthographic neighbors, and frequency of those orthographic neighbors. In addition, Jalbert et al. measured output time and confirmed this was equivalent for short and long words. In both experiments, there was no effect of word length. In a follow-up study, Jalbert et al. (2011b) used nonwords instead of words because they allow for a 2 × 2 factorial design that manipulated length (short vs. long) and neighborhood size (small vs. large). They found a main effect of neighborhood size, no effect of length, and no interaction.

There are a number of potential objections to the two experiments reported by Jalbert et al. (2011a, b). First, although Jalbert et al. tested two different stimulus sets, both were relatively small, and as we have noted previously, small stimulus sets can show unusual results that do not generalize. Second, although Jalbert et al. equated the short and long words on more dimensions than previous researchers, there are still many dimensions that were not equated, including measures of phonological neighborhood size and frequency. Third, the words were not equated on structural typicality; that is, they were not equated for constrained and unconstrained unigram, bigram, and trigram counts. A constrained unigram is a specific letter in a specific position in a word of a specific length. For example, the *o* in *stoat* is considered the same as the *o* in fl*o*at (same position, same length word) but is not considered the same as the *o* in *coach* (same length word but different position) or the *o* in vi*o*lin (same position but different length). An unconstrained unigram allows the letter to be in any position of a word of the same length. The same definitions hold for bigrams and trigrams except these count two or three adjacent letters, respectively. However, a simple count does not address the fact that these measures covary with length. Storkel (2004) examined three different ways of removing the correlation between these measures and length and concluded that computing *z* scores was most appropriate.

Guitard et al. (2018) addressed all three issues. First, they created two larger stimulus sets, one with 30 one-syllable and 30 three-syllable words, and the other with 36 two-syllable and 36 four-syllable words. Second, they equated the short and long words on 17 dimensions, including phonological neighborhood size and frequency. Third, they also considered structural similarity by equating the short and long words on constrained and unconstrained unigram, bigram, and trigram counts. Importantly, they used measures recommended by Storkel (2004) which removes the correlation with length. Like Jalbert et al. (2011a, b), Guitard et al. also measured output time to confirm that it was equivalent for lists of short and long items. Once again, no word-length effects were observed.

## Word length and serial recognition

In a serial recall test, a short list of items is shown one at a time for about a second each and then the person is asked to recall the items in order, either by speaking, writing, typing, or clicking on appropriately labelled buttons. A serial recognition task begins the same way but after presentation of the first list, a second list is presented that contains the same items. On half of the trials, the second list is the same as the first but on the other half of the trials two adjacent items are transposed. The task is to indicate whether the words in the two lists are in the same order or a different order.

To our knowledge, there are only two published studies that have looked at word-length effects in serial recognition despite the latter task having a number of potential advantages for studying the word-length effect. First, only one response is made on each trial, clicking on a button or pressing a key to indicate a same or different response. This is more likely to equate output time than having multiple responses. Second, because only one response is made, output interference is also equated. Third, Baddeley et al. (2002) have suggested that relative to serial recall, serial recognition is less likely to be sensitive “to slight differences in the characteristics of the long and short words” (p. 357). This is based on other results that manipulated dimensions such as lexicality, whether a stimulus is a word or nonword. For example, Gathercole et al. (2001) found a large effect of lexicality in serial recall—the proportion correct for lists of words was 0.72 compared with 0.42 for lists of nonwords—but only a small effect in serial recognition—the proportion correct was 0.87 versus 0.80, respectively. Indeed, a common view is that serial recognition “provides a relatively pure estimate of phonological short-term memory” uncontaminated by long-term memory factors (Gisselgård et al., 2007, p. 358; see also Thorn et al., 2002). Together, these characteristics mean that serial recognition is likely an excellent method to test the standard model: Explanations other than the standard model, such as differential output time and interference or differences on dimensions that covary with length, should predict no word-length effect because of the type of test. In contrast, the standard model holds that items should still decay during presentation and articulatory rehearsal is still necessary to offset this decay. Because of this, word-length effects should obtain.

Baddeley et al. (2002) reported three experiments that used serial recognition and manipulated word length. They used lists constructed from a small fixed pool of 10 one-syllable words (*cheese, crab, ear, eye, ski, stool, sun, tent, toad, torch*) and 10 three-syllable words (*caravan, celery, elephant, envelope, screwdriver, strawberry, submarine, telephone, tomato, typewriter*). They purposely chose words with overlapping initial letters to prevent subjects from using a strategy of remembering only the initial letter. However, because the experiments were conducted prior to the widespread availability of norms, the short and long words were equated on only five dimensions. This should not necessarily be an issue, though, if the presumed relative insensitivity of serial recognition to lexical factors is correct. Baddeley et al. consistently found better performance for lists of short words than lists of long words. In their Experiment 3, for example, 16 undergraduates experienced 20 lists of each type, short or long. Estimating from their Fig. 5, the proportion correct for lists of short words was 0.86 compared with 0.74 for lists of long words. Baddeley et al. concluded that the data support the standard model. One potential weakness, however, is that only one stimulus set was used, compounded by the fact that there were only 10 words of each kind.

Campoy (2008) also looked at word-length effects in serial recognition and also used a single small fixed set of stimuli—in this case, a set of eight two-syllable and eight three-syllable Spanish words. The short and long words were equated on only five dimensions. Unlike the Baddeley et al. (2002) studies, the experiments were designed to assess the role of rehearsal by including conditions that minimized opportunities to rehearse. For example, in Experiment 1, 25 undergraduates saw words presented very quickly at a rate of one word every 300 ms, making rehearsal during presentation unlikely. In this condition, performance (measured by *A′*) was better for lists of short words than for lists of long words, 0.85 versus 0.76, respectively. In all three experiments, whether with visual or auditory presentation, there was a word-length effect regardless of whether rehearsal was likely or unlikely. This latter result poses a problem for the standard model. As Campoy noted, if rehearsal is minimized, then the standard model predicts equivalent performance for short and long words because they both decay at the same rate. It is because of this lack of an effect of rehearsal opportunity that Campoy interpreted the results in terms of retroactive interference, as noted previously.

There are a number of reasons to reexamine the results of both studies. First, both studies used a single small set of words. In addition to the time-based word-length effect studies, there are a number of other examples of experiments involving short and long words that differ across different pool sizes. For example, Cowan et al. (2003) and Hulme et al. (2004) found different results when mixing short and long words within the same list. However, Cowan et al. used a small pool of words (six short and six long) whereas Hulme et al. used a large pool of words (80 short and 80 long). Bireta et al. (2006) replicated the results of Cowan et al. when using their pool and replicated the different results of Hulme et al. when using their pool. Although the small pool produced consistent results, these did not generalize to a larger pool. Small and large pools may yield different results for a number of reasons. One reason may be that a small sample may be less representative of words in general than a large sample. A second is that a single unusual item in a small pool could be sufficient to affect the result whereas the same unusual item in a large pool would likely have no effect. A third reason, suggested by LaPointe and Engle (1990), may be that when people see the same words on multiple trials, they use different processing than when the words occur less often. One purpose of the current work, then, is to use both small and large pools.

A second reason to reexamine these results is that although the short and long words in each set of experiments were equated on five dimensions, many new norms and databases have become available. For example, there are now online databases of dimensions that covary with length, including orthographic and phonological neighborhood information for both English (e.g., Balota et al., 2007) and Spanish (e.g., Marian et al., 2012). In particular, the short words used by Baddeley et al. (2002) have a mean of 7.2 orthographic and 15.30 phonological neighbors compared with 0.30 orthographic and 0.30 phonological neighbors for the long words. The words used by Campoy (2008) also differ in orthographic and phonological neighborhood size: The mean number of orthographic neighbors was 9.75 for the short words compared with 1.29 for the long words; the corresponding values for phonological neighbors is 12.25 and 1.43.^1^ Will the word-length effect remain if the short and long words are equated on these dimensions?

A third reason is simply to obtain further data. Unlike serial recall, serial recognition has not been extensively studied and it is not a given that an effect routinely observed in serial recall will be observed in serial recognition. For example, Chubala et al. (2019) found semantic relatedness effects—better performance with lists of words from the same category compared with lists of words from different categories—in serial recall but not in serial recognition (see also Murdock, 1976). This is consistent with the view discussed previously that serial recognition may be relatively insensitive to lexical/long-term memory factors. It may be the case that whereas neighborhood size and frequency effects are robust in serial recall, they may be absent in serial recognition. Of the two studies examining neighborhood factors in serial recognition, Greeno et al. (2022) found no effect, whereas Guitard et al. (2024) did find a neighborhood size effect. Additional data would add more clarity to this discrepancy.

Experiment 1 was designed as a conceptual replication of Baddeley et al. (2002) and Campoy (2008) in that it used short and long words that also differed on dimensions that covary with length. All accounts—whether based on the standard model, retroactive interference, or lexical/long-term memory factors—predict a word-length effect. We included both a small stimulus set as well as a large set to assess any effects of set size.

Experiment 2 was designed to compare predictions of the three accounts. We used a new set of stimuli that were equated on 17 dimensions, including both orthographic and phonological neighborhood size and frequency. The standard model predicts a word-length effect because the items will decay which will need to be offset by rehearsal. More short words can be rehearsed than long words, leading to an advantage for lists with short words. The interference account predicts a word-length effect because the short and long words differ in length, just as they did in Campoy’s (2008) study. However, the lexical/long-term memory factors’ account predicts no difference because the short and long words are more fully equated on the critical dimensions.

Experiment 3 was designed to verify that lexical/long-term memory factors affect serial recognition, in contrast to the assumption made by Baddeley et al. (2002). We created a new set of stimuli in which the words were equated for length but varied in neighborhood size and frequency. Because the words in the two conditions are equated for length, neither the standard model nor the interference account predict a difference, although neither includes the manipulated dimensions within their scope. In contrast, this is a key test of the lexical/long-term memory factors account because it predicts better performance for words with more orthographic and phonological neighbors.

## Experiment 1

### Method

The experiment was an amalgam of the methods used by Baddeley et al. (2002) and Campoy (2008) with some modifications. First, in addition to a small fixed set of stimuli, we also included a large set and a small random set. For the latter, 10 short and 10 long words were randomly drawn from the larger pool for each subject. On average, each subject in this condition received a different small set of items. The reason for this small random condition is to control for any idiosyncratic effects that might exist in a single small fixed set of stimuli. Second, we used only visual presentation because it allows for more simple stimulus manipulation and presentation and because Campoy included both visual and auditory presentation and observed the same pattern of results for each. Third, Baddeley et al. randomly chose the two adjacent items to transpose whereas Campoy ensured equal transpositions of all pairs. We followed Campoy but added the constraint that the first item was never transposed. Finally, we used five-item lists like Campoy rather than six-item lists like Baddeley et al.

#### Ethics

The research was approved by Cardiff University’s School of Psychology Ethics Committee.

#### Sample size

We used the fixed-*n* version of Bayes factor design analysis (Schönbrodt & Wagenmakers, 2018) to estimate the smallest sample size that would be likely to provide informative Bayes factors. The key statistical comparison is a Bayesian within-subjects *t* test comparing *d′* for short and long words. We expect the effect size in Experiment 1 to be larger than that in Experiment 3 because the stimuli differ on more dimensions. Because we want the same sample size in all three studies, we used effect sizes associated with neighborhood size effects. Guitard et al. (2024) reported an effect size of *d* = 0.477 and 0.462 when looking at neighborhood size effects in serial recognition with a large and small pool, respectively. We therefore used an effect size of *d* = 0.462 for the alternative hypothesis. For the null hypothesis, we used an effect size of *d* = 0.0. The decision boundary was set at BF > 3.0. For each hypothesis, 10,000 simulations were run that calculated a non-directional Bayesian within-subjects *t* test using the BFDA package (Schönbrodt & Stefan, 2019). For the alternative hypothesis, the simulations indicated that with 70 subjects 88.9% of the samples indicated evidence for the alternative hypothesis (BF > 3), 10.4% were inconclusive (0.333 < BF < 3), and 0.7% indicated evidence for the null hypothesis (BF < 0.333). For the null hypothesis, simulations indicated that 84.0% of the samples showed evidence for the null hypothesis (BF < 0.333); 15.1% were inconclusive (0.333 < BF < 3), and 0.9% showed evidence for the alternative hypothesis (BF > 3). Based on these simulations, we decided on a sample size of 70 in each between-subject condition because it should be unlikely to result in uninformative Bayes factors.

#### Subjects

A total of 210 native speakers of English were recruited from Cardiff University in exchange for course credit and were randomly assigned to one of three conditions. The mean age was 19.44 years (*SD* = 2.38); 182 self-identified as female, 25 as male, and three as other.

#### Design

Set size (small fixed, small random, or large) was manipulated between subjects, whereas length (short or long) was manipulated within subjects.

#### Stimuli

The small fixed set used the 10 short and 10 long words from Baddeley et al. (2002). In addition to differing in length (one syllable vs. three syllable), they also differed on various measures of orthographic and phonological neighborhood factors, as well as differing in frequency and contextual diversity (using the Brysbaert & New, 2009, norms). The large set had 60 short and 60 long words and was created by selecting one- and three-syllable words that were similar to the small set, although there was no attempt to match the two sets exactly. Summary details are in Table 4, and the full set of stimuli for all experiments are available from the Open Science Foundation (10.17605/osf.io/68ye4).

#### Procedure

After reading a consent form and agreeing to participate, the subjects were randomly assigned to one of the three set-size conditions. Each trial began when the subject clicked on a button labelled “Start next trial.” Five words were randomly drawn from the appropriate pool (i.e., short or long) and were shown one at a time for 1 s in the center of the screen in 28-point Helvetica. Two s after the final word had been shown, a second list was shown at the same rate. Half the time this second list was identical to the first and half the time two adjacent items were transposed. A message then appeared prompting the subject to indicate whether the order of the words was the same or different and they responded by clicking on an appropriately labelled button. Subjects were informed that half the time the lists would be identical and that half the time two adjacent items would be transposed.

There were 60 trials. Half the trials had short words and half had long words. For each type of list, there were 15 same and 15 different trials. For different trials, Words 2 and 3, 3 and 4, and 4 and 5 were transposed equally often; the first word was never transposed. The order of these trials was randomly determined for each subject. For the large pool condition, words were randomly sampled without replacement from the appropriate pool for each trial. After 12 trials of a given condition (i.e., short or long), all 60 words would have been used from that pool. At this point, the pool was restored to 60 words and sampling without replacement began again. Thus, each word appeared either two or three times during the experiment. For the small random condition, 10 short and 10 long words were randomly sampled from the larger pool for each subject. On each trial, five short or five long words were randomly sampled from the small pool. For the small fixed condition, five short or five long words were randomly sampled from the Baddeley et al. (2002) stimulus set.

#### Data analysis

The data were analyzed using JASP (JASP Team, 2024), and we report a Bayes factor, BF_10_, that indicates evidence for the alternative hypothesis. We interpret a value between 3 and 10 as indicating substantial evidence; a value between 10 and 30 indicating strong evidence; values between 30 and 100 indicating very strong evidence; and values greater than 100 indicating decisive evidence (Wetzels et al., 2011). BF_01_ indicates evidence for the null hypothesis using the same scale.

A hit was defined as correctly responding “different” to a different list, and a false alarm was defined as incorrectly responding “different” to a same list. These values were transformed as recommended by Snodgrass and Corwin (1988) to prevent hit or false alarm rates of 1 or 0. The transformed hit rate is calculated according to the formula $\frac{\#H + 0.5}{\#D + 1}$, where #H is the number of hits and #D is the number of different trials. The false alarm rate was calculated similarly. From these transformed hit and false alarm rates we calculated *d′*, the ability to discriminate between same and different trials, and *C*, a measure of response bias (see Macmillan & Creelman, 2005).

### Results and discussion

**Fig. 1:**
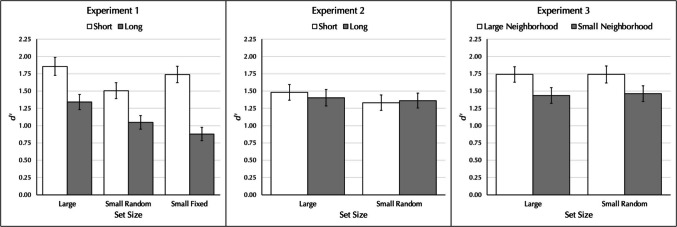
Performance, *d′*, for short and long words that were not well equated as a function of set size in Experiment 1 (left panel); for short and long words that were more fully equated in Experiment 2 (middle panel); and for words equated for length but which differed in the size and frequency of their orthographic and phonological neighborhoods in Experiment 3 (right panel). Error bars show the standard error of the mean

Table 1 shows the hit and false alarm rates, *d′*, and *C* for the short and long words, as well as the effect size and the Bayes factors for a within-subjects *t* test comparing the short and long conditions on each measure. As can also be seen in the left panel of Fig. 1, there was a word-length effect in each of the three set-size conditions, with the largest word-length effect obtained using the small fixed set from Baddeley et al. (2002). The effect size of *d* = 1.073 for the small fixed set was almost twice the size of the other two conditions, 0.557 for the large and 0.563 for the small random. Finding a word-length effect in serial recognition with confounded stimuli replicates the results reported by Baddeley et al. (2002) and Campoy (2008) and is the pattern predicted by all accounts. Moreover, the effect was observed for all three set-size conditions, though substantially larger for the small fixed set.

**Table 1:** Performance measures for short and long words for each set-size condition in Experiment 1, and the effect size and Bayes factor comparing short and long words on each measure

	Short Words		Long Words			
	Large Set					
	*M*	*SD*	*M*	*SD*	Cohen’s *d*	BF_10_
Hit	0.761	0.174	0.686	0.176	0.449	64.498
FA	0.193	0.158	0.246	0.159	0.362	8.449
*d′*	1.856	1.084	1.344	0.909	0.557	1203.201
*C*	−0.087	0.308	−0.116	0.325	0.081	0.136
	Small Random Set					
	*M*	*SD*	*M*	*SD*	Cohen’s *d*	BF_10_
Hit	0.738	0.161	0.671	0.163	0.444	56.436
FA	0.252	0.147	0.312	0.160	0.370	10.054
*d′*	1.505	0.954	1.048	0.810	0.563	1434.606
*C*	−0.015	0.290	−0.029	0.293	0.039	0.138
	Small Fixed Set					
	*M*	*SD*	*M*	*SD*	Cohen’s *d*	BF_10_
Hit	0.754	0.170	0.604	0.192	1.011	2.90 × 10^9^
FA	0.209	0.145	0.310	0.159	0.648	1.82 × 10^4^
*d′*	1.740	0.987	0.878	0.800	1.073	2.44 × 10^10^
*C*	−0.059	0.327	−0.130	0.407	0.190	0.436

## Experiment 2

### Method

Experiment 1 found a word-length effect with all three set-size conditions using stimuli in which word length was confounded with other measures including orthographic and phonological neighborhood size. The purpose of Experiment 2 was to assess whether a word-length effect would obtain if the short and long words were more fully equated. The standard model and the interference account both predict a word-length effect despite being more fully equated whereas the lexical/long-term memory factors account predicts no word-length effect. We omitted the small fixed set-size condition, leaving only two between-subject conditions: large versus small randomized set size.

#### Subjects

One hundred and forty different native speakers of English were recruited from Cardiff University in exchange for course credit and were randomly assigned to one of two conditions. The mean age was 19.22 years (*SD* = 1.84); 116 self-identified as female, 23 as male, and one as other.

#### Design

Set size (small random or large) was manipulated between subjects, whereas length (short or long) was manipulated within subjects.

#### Stimuli

A new set of stimuli were created in which there were 60 short (two syllable) and 60 long (three syllable) words. The short and long words were equated on 19 dimensions, including frequency, contextual diversity, orthographic and phonological neighborhood size and frequency, concreteness, prevalence, semantic density, semantic neighborhood size, semantic diversity, age of acquisition, valence, arousal, and dominance. The words differed in number of phonemes (*M* = 4.95, range: 4–5 for short versus *M* = 7.2, range: 6–9 for long), number of letters (*M* = 6.55, range: 6–7 for short versus *M* = 8.7, range: 8–10 for long), and number of syllables (two vs. three). Details are shown in Table 5.^2^ For the small random set size condition, 10 short and 10 long words were randomly selected for each subject.

#### Procedure

Except for the stimuli and omitting the small fixed condition, the procedure was the same as in Experiment 1.

### Results and discussion

Table 2 shows the hit and false alarm rates, *d′*, and *C* for the short and long conditions, as well as the effect size and Bayes factors comparing the two conditions on each measure. As can also be seen in the middle panel of Fig. 1, there was no difference in performance as a function of length. The Bayes factors all indicate evidence for the null hypothesis. This result with serial recognition parallels earlier findings with serial recall that the word-length effect disappears when the short and long words are equated on more dimensions and especially for orthographic and phonological neighborhood size and frequency (e.g., Guitard et al., 2018; Jalbert et al., 2011a, b). The results are as predicted by the lexical/long-term memory account and are contrary to both the standard model and the interference account: Both predict a word-length effect, the former because more short words can still be rehearsed compared with long words and the latter because there is still differential interference.

**Table 2:** Performance measures for short and long words for each set size condition in Experiment 2, and the effect size and Bayes factor comparing short and long words on each measure

	Short Words		Long Words			
	Large Set					
	*M*	*SD*	*M*	*SD*	Cohen’s *d*	BF_01_
Hit	0.696	0.182	0.677	0.187	0.141	3.907
FA	0.214	0.133	0.226	0.138	0.108	5.156
*d′*	1.482	0.931	1.404	0.989	0.125	4.499
*C*	−0.146	0.305	−0.156	0.298	0.032	7.363
	Small Random Set					
	*M*	*SD*	*M*	*SD*	Cohen’s *d*	BF_01_
Hit	0.728	0.168	0.715	0.181	0.079	6.159
FA	0.287	0.152	0.275	0.166	0.081	6.116
*d′*	1.333	0.912	1.361	0.893	0.036	7.285
*C*	0.027	0.290	−0.003	0.393	0.082	6.071

Unlike Table 1, Table 2 reports BF_01_ rather than BF_10_.

If we are to claim that lexical factors including orthographic and phonological neighborhoods are involved in producing word-length effects, it is necessary to demonstrate such effects in serial recognition using similarly highly controlled stimuli. It is not obvious that orthographic and phonological neighborhood effects will be found for two reasons. As noted previously, not all effects observed in serial recall are found in serial recognition. For example, semantic relatedness effects are readily observed in serial recall but are absent in serial recognition (Chubala et al., 2019; Murdock, 1976). This absence of a semantic relatedness effect is consistent with claims that unlike serial recall, serial recognition “provides a relatively pure estimate of phonological short-term memory” and is therefore insensitive to lexical or long-term memory factors (Gisselgård et al., 2007, p. 358; see also Thorn et al., 2002). If this view is correct, then neighborhood size effects may be absent, just like semantic relatedness effects.

Second, the two studies that have looked at neighborhood effects in serial recognition found different results despite using the same stimuli. Greeno et al. (2022) reported two experiments, one with a large pool and one with a small fixed pool. They found no effect for the large pool and an advantage for small neighborhood size lists for the small pool; in serial recall, there is an advantage for large neighborhood size in both small and large pools. However, their experimental design had two aspects that may have contributed to the results. First, rather than randomly generating each list for each subject, all subjects received the same lists. The problem with using the same lists for all subjects is that if by chance some of the lists differ from others, the observed result may be due to an unwanted confound. Consider the following an example.^3^ Two pools of words, A and B, are equated for mean frequency. The words from each pool are then randomly assigned to 10 lists, five of each type. If the mean frequency of each list is calculated and then rank ordered, it is possible that Pool A has more higher frequency lists than Pool B. Frequency becomes a confound and the Pool A lists are remembered better, but not because the A and B pools differ on the dimension of interest. Something like this could explain the results for the large pool experiment. The second potential issue is that only one small pool was tested and as we have previously discussed small pools may produce atypical results. Guitard et al. (2024) reported two experiments that addressed these issues. They used the same stimuli as Greeno et al. (2022) but used randomly generated lists for everyone. In the first experiment, they found the usual memory advantage for large neighborhood words with the large pool. In the second study, they randomly generated a small pool of 12 large and 12 small neighborhood words for each subject and observed a neighborhood size effect. Nonetheless, only one set of stimuli has been tested. Given this, we deemed it necessary to replicate their study using a new set of stimuli.

## Experiment 3

### Method

Experiment 2 found that when short and long words were equated on many dimensions, including orthographic and phonological neighborhood size and frequency, the word-length effect observed in Experiment 1 disappeared. However, it is necessary to show that when words are equated for length, orthographic and phonological neighborhood factors affect serial recognition. The problem is that only two studies have examined this but they obtained different results. Therefore, Experiment 3 was designed as a conceptual replication of Guitard et al. (2024). Set size, large versus small, was a between-subjects manipulation and neighborhood size was a within-subjects manipulation. Importantly, we used a new set of stimuli. The key prediction from the lexical/long-term memory factors account is that performance will be better for words with more neighbors than words with fewer neighbors. In contrast, accounts which suggest serial recognition is not sensitive to lexical and long-term memory factors predict no effect (e.g., Baddeley et al., 2002; Gathercole et al., 2001; Gisselgård et al., 2007).

#### Subjects

One hundred and forty different native speakers of English were recruited from Cardiff University in exchange for course credit and were randomly assigned to one of two conditions. The mean age was 19.11 years (*SD* = 0.97) ; 122 self-identified as female, 16 as male, and one as other.

#### Design

Set size, small random or large, was manipulated between subjects, whereas neighborhood size, small or large, was manipulated within subjects.

#### Stimuli

A new set of stimuli were created that differed in neighborhood size measures but were equated on other dimensions including word length (number of phonemes, letters, and syllables). The large neighborhood words had a mean of 7.83 orthographic neighbors compared with 0.22 for the small. They also had a mean of 12.50 phonological neighbors compared with 1.03 for the small. They also differed in OLD (1.48 vs. 2.27) and PLD (1.33 vs. 2.02). There were a total of 72 words, 36 in each group. Details are shown in Table 5.

#### Procedure

Other than the change from manipulating word length to manipulating neighborhood size, and the corresponding use of different stimuli, the procedure was the same as in Experiment 2.

### Results and discussion

Table 3 shows the hit and false alarm rates, *d′*, and *C* for the large and small neighborhood size conditions, as well as the effect size and Bayes factors comparing the two conditions on each measure. As can also be seen in the right panel of Fig. 1, there was a neighborhood size effect for each set size. The results replicate those of Guitard et al. (2024) but using a different stimuli. This generalization provides additional evidence to support the idea that the unusual results reported by Greeno et al. (2022) were due to methodological factors. In addition, the finding of a large neighborhood size advantage in serial recognition questions the assumption of some accounts that serial recognition is not sensitive to such factors (e.g., Baddeley et al., 2002; Gathercole et al., 2001; Gisselgård et al., 2007). More importantly, these findings are consistent with the idea that orthographic and phonological neighborhood differences may be one of the lexical factors driving the word-length effect in serial recognition, just as it does in serial recall.

**Table 3:** Performance measures for large and small neighborhood words for each set size condition in Experiment 3, and the effect size and Bayes factor comparing large and small neighborhood words on each measure

	Large Neighborhood		Small Neighborhood			
	Large Set					
	*M*	*SD*	*M*	*SD*	Cohen’s *d*	BF_10_
Hit	0.754	0.166	0.717	0.166	0.332	4.512
FA	0.205	0.135	0.246	0.143	0.336	4.863
*d′*	1.741	0.926	1.436	0.947	0.466	100.144
*C*	−0.067	0.329	−0.055	0.263	0.041	0.139
	Small Random Set					
	*M*	*SD*	*M*	*SD*	Cohen’s *d*	BF_10_
Hit	0.757	0.161	0.735	0.156	0.180	0.384
FA	0.214	0.138	0.255	0.137	0.354	7.059
*d′*	1.741	1.013	1.464	0.932	0.408	23.621
*C*	−0.046	0.305	−0.013	0.258	0.102	0.186

## General discussion

Three experiments reexamined whether length causes the word-length effect reported in serial recognition. Experiment 1 replicated the results of Baddeley et al. (2002) and Campoy (2008) that short words led to better performance than long words with a small fixed set of stimuli, and extended this result to both a small random and a large set size. Importantly, the short and long words also differed on a number of other dimensions that covary with length including orthographic and phonological neighborhood size and frequency. Experiment 2 used a new set of stimuli in which short and long words were equated on more dimensions, including orthographic neighborhood size and frequency, and the word-length effect observed in Experiment 1 disappeared; the Bayes factors indicated substantial evidence for the null hypothesis. Experiment 3 compared serial recognition of words that were equated for length but which had either a large or a small orthographic/phonological neighborhood and found a large neighborhood advantage for both a large pool and a small pool, replicating results reported by Guitard et al. (2024). This is contrary to the idea that serial recognition is insensitive to lexical and other long-term memory factors.

The results are problematic for accounts that are based on the standard model in which decay is offset by rehearsal. According to these accounts, items in temporary storage decay unless the decay is offset by rehearsal. On the assumption that rehearsal rate is correlated with the length of the word, more short words can be maintained than long words. This account predicts the word-length effect seen in Experiment 1, but it also predicts a word-length effect in Experiment 2. According to the standard model, rehearsal should still take longer for long than short words even when the words are equated for neighborhood size. The results are also problematic for the interference account. According to this view, long words produce more retroactive interference than short words, and therefore a word-length effect should have been observed in Experiment 2. The results are as predicted by the lexical/long-term memory factors account. On this view, the word-length effect seen in Experiment 1 was due to confounding variables. When the words were more fully equated in Experiment 2, the word-length effect disappeared. When the words were equated for length but differed in orthographic and phonological neighborhood factors in Experiment 3, there was an advantage for large neighborhood words.

It might be objected that the manipulation in Experiment 2, comparing two- and three-syllable words, may not have been large enough to produce a word-length effect. Although possible, we think this is unlikely. Campoy (2008) found word-length effects in serial recognition when comparing two- and three-syllable words, and many studies have reported word-length effects when comparing two- and three-syllable words using serial recall (e.g., Baddeley et al., 1975, Exp. 6; Hulme & Tordoff, 1989, Exp. 1; Guitard et al., 2018, Exp. 3; McNeil & Johnston, 2008, Exp. 3; Romani et al., 2005, Exp. 1). We think a more likely explanation is that the short and long words in Experiment 2 were more fully equated thus removing confounds present in other studies.

A second potential objection may be that there are numerous dimensions that we did not control, and it may be that our stimuli have confounds that drove our results. That is, some as yet unidentified confound in Experiment 2 favored the long words over the short and cancelled out the usual short-word advantage. As we have noted in previous work, this is ultimately an empirical question and is easy to test: Researchers can create a new set of stimuli in which the short and long word pools are equated on more dimensions than we did and can then assess whether the word-length effect reappears. If it does, it would suggest there was an unidentified confound in our stimuli but if it does not, it would be additional evidence in favor of the lexical/long-term memory factors account.

Experiment 3 confirmed that at least some lexical/long-term memory factors are observed in serial recognition, contrary to suggestions that serial recognition is not affected by such factors (e.g., Baddeley et al., 2002; Gathercole et al., 2001; Gisselgård et al., 2007). Serial recognition remains rather understudied compared with other methods of testing, and it is not yet clear which lexical or long-term memory factors will be observed and which will not. Nonetheless, the finding of neighborhood effects in serial recognition means that descriptions of serial recognition as being “as close to a pure order task as possible” (Thorn et al., 2002, pp. 313–314) are not tenable.

How do orthographic and phonological neighborhood characteristics affect short-term/working memory tasks such as serial recognition? With the caveat that there is insufficient data to determine whether other factors that covary with length may also be involved, here is one possible account. According Roodenrys’s (2009) redintegration framework as applied to serial recall, as each word is encountered, it can serve as input to an interactive activation network. Each word will partially activate its orthographic and phonological neighbors, and words with more neighbors will partially activate more items than words with fewer neighbors. The activation from the neighbors feeds back to the list item and because of this, words with more neighbors will receive more feedback activation than words with fewer neighbors. Because short words tend to have more neighbors than long words (Sears et al., 2006), short words will receive more feedback activation and therefore will be remembered better than long words. If the words are equated on these lexical dimensions, then the differential activation is removed, and the word-length effect disappears.

How exactly does feedback activation boost performance for large neighborhood words in serial recognition? To our knowledge, there is only one model of serial recognition, that of Farrell and McLaughlin (2007), and although it did not specify how factors such as neighborhood size would affect serial recognition, we think Roodenrys’s (2009) suggestion can be readily incorporated into the model. Farrell and McLaughlin proposed that items are represented by their time of encoding and these temporal values drift over time. When the second list is presented, the first list will be temporally noisy compared with the second. A decision to respond same or different is based on an overall difference score that compares the two lists. When this difference exceeds a criterion, a “different” judgment is made; when it fails to reach the criterion, a “same” judgment is made. The model does not specifically include word length or any lexical/long-term memory factors, but as Chubala et al. (2019) reasoned, the calculation of an overall difference score occurs without redintegration: The representations of the items are compared without having to identify (or redintegrate) each individual item. The general prediction is that serial recognition should show effects that do not require redintegration, such as acoustic similarity, but should not show effects that do require redintegration, such as semantic relatedness. This is the pattern of results Chubala et al. observed.

On this account, it may be the case that the feedback activation, such as that proposed by Roodenrys (2009), leads to less noisy representations for items that receive more feedback compared with items that receive less feedback, and therefore the difference score for large neighborhood trials would be, on average, smaller than for small neighborhood trials. In other words, the difference score should be more accurate for lists of large neighborhood words than for lists of small neighborhood words. Although implementation of this idea within the model is beyond the scope of the current work, it does make the general prediction that serial recognition will be affected by any manipulation that increases feedback activation.

## Summary

The results of three experiments in serial recognition are consistent with similar studies using serial recall: Word-length effects are observed when the short and long words also differ on lexical dimensions including phonological and orthographic neighborhood measures but are not observed when the short and long words are more fully equated. The results are problematic for any account based on the standard model, where decay is offset by rehearsal, and are also problematic for accounts based on retroactive interference, because more fully equated long and short words still differ in length. The results are consistent with a growing body of work that shows that lexical and other long-term memory factors affect short-term/working tasks. As such, the results provide even more evidence that short-term/working memory tasks are always subject to contamination by lexical and long-term memory factors.

## Appendix

Tables 4 and 5

**Table 4:** Characteristics of the words in the small fixed pool (left, words from Baddeley et al., 2002) and words in the large pool used in Experiment 1 and a *t* test comparing the short and long words on each measure

	Small Pool						Large Pool					
	Short		Long				Short		Long			
	*M*	*SD*	*M*	*SD*	*t*	*p*	*M*	*SD*	*M*	*SD*	*t*	*p*
LgHAL	8.70	1.48	7.97	1.23	1.19	.25	7.36	1.07	7.60	1.46	1.00	.32
LgSubTLWF	2.91	0.52	2.44	0.41	2.24	.04	2.19	0.43	2.12	0.50	0.79	.43
LgSubTLCD	2.68	0.51	2.21	0.39	2.33	.03	2.00	0.41	1.97	0.46	0.29	.77
Ortho_N	6.80	5.69	0.00	0.00	3.78	.00	6.37	5.25	0.00	0.00	9.40	.00
Phono_N	15.30	9.73	0.30	0.67	4.86	.00	13.75	9.77	0.00	0.00	10.90	.00
OLD	1.51	0.38	3.37	0.79	6.71	.00	1.58	0.32	2.75	0.15	25.96	.00
OLDF	8.46	0.68	6.51	0.49	7.37	.00	8.12	0.59	7.09	0.41	11.19	.00
PLD	1.27	0.33	3.41	0.97	6.59	.00	1.34	0.32	2.84	0.47	20.18	.00
PLDF	8.97	1.10	6.18	0.87	6.30	.00	8.33	0.92	7.08	0.61	8.78	.00
Conc	4.86	0.13	4.86	0.19	0.08	.93	4.32	0.75	3.42	0.91	5.87	.00
SemDen	0.57	0.07	0.52	0.09	1.44	.17	0.50	0.10	0.53	0.10	1.87	.06
SemNeigh	2,442.80	3,093.40	1,260.50	2,070.02	1.00	.33	596.85	1,559.70	1,806.53	2,376.85	3.30	.00
SemDiv	1.53	0.20	1.35	0.33	1.43	.17	1.44	0.22	1.48	0.32	0.77	.44
AoA	5.24	1.47	6.02	1.62	1.13	.27	8.71	2.52	9.81	1.94	2.67	.01
Valence	5.87	0.82	5.97	0.49	0.32	.75	5.13	0.90	5.01	1.41	0.54	.59
Arousal	4.06	1.01	3.51	0.75	1.37	.19	3.88	0.58	4.35	0.87	3.43	.00
Dominance	5.57	0.86	5.60	0.75	0.07	.94	5.26	0.66	4.92	1.06	2.13	.04
NPhon	3.10	0.99	7.20	0.92	9.58	.00	3.65	0.78	7.20	0.90	23.15	.00
NLet	4.00	1.05	8.40	1.71	6.92	.00	4.40	0.94	8.70	0.77	27.43	.00
NSyll	1.00	0.00	3.00	0.00	–	–	1.00	0.00	3.00	0.00	–	–
Prev	2.26	0.13	2.35	0.19	1.27	.22	2.07	0.34	2.28	0.22	4.06	.00
PKnown	1.000	0.000	0.996	0.011	1.000	.331	0.975	0.036	0.992	0.016	3.226	.002

LgHAL = log HAL frequency (from Balota et al., 2007); LgSubTLWF = log subtitle word frequency; LgSubTLCD = log subtitle contextual diversity (both from Brysbaert & New, 2009); Ortho_N = number of orthographic neighbors; Phono_N = number of phonological neighbors (both from Balota et al., 2007); OLD = orthographic Levenshtein distance; OLDF = frequency of neighbors defined by OLD; PLD = phonological Levenshtein distance; PLDF = frequency of neighbors defined by PLD (all four measures from Yarkoni et al., 2008); Conc = concreteness rating (from Brysbaert et al., 2014); SemDen = semantic neighborhood density; SemNeigh = semantic neighbors (both from Shaoul & Westbury, 2010); SemDiv = semantic diversity (Hoffman et al., 2013); AoA = age of acquisition (Kuperman et al., 2012); Valence, Arousal, and Dominance ratings (from Warriner et al., 2013); NPhon = number of phonemes; NLet = number of letters; NSyll = number of syllables (from Balota et al., 2007); Prev = prevalence (from Brysbaert et al., 2019); PKnown = proportion of words known (from Brysbaert et al., 2014)

**Table 5:** Characteristics of the words in the pool used in Experiment 2 (left panel) and Experiment 3 (right panel) and a *t* test comparing the short and long words (left panel) and large and small neighborhood words (right panel) on each measure

	Experiment 2						Experiment 3					
	Short		Long				Large		Small			
	*M*	*SD*	*M*	*SD*	*t*	*p*	*M*	*SD*	*M*	*SD*	*t*	*p*
LgHAL	7.41	1.30	7.60	1.46	0.74	.46	7.85	1.42	8.08	1.44	0.69	.49
LgSubTLWF	2.06	0.45	2.12	0.50	0.75	.46	2.36	0.57	2.32	0.60	0.31	.76
LgSubTLCD	1.90	0.43	1.97	0.46	0.88	.38	2.18	0.54	2.17	0.53	0.06	.95
Ortho_N	0.02	0.13	0.00	0.00	1.00	.32	0.22	0.42	7.83	1.96	22.73	.00
Phono_N	0.00	0.00	0.00	0.00	–	–	1.03	1.03	12.50	4.42	15.18	.00
OLD	2.73	0.31	2.75	0.15	0.59	.56	2.27	0.32	1.48	0.14	13.53	.00
OLDF	7.06	0.39	7.09	0.41	0.45	.65	7.27	0.50	7.47	0.46	1.76	.08
PLD	2.71	0.40	2.84	0.47	1.61	.11	2.02	0.26	1.33	0.24	11.70	.00
PLDF	7.04	0.72	7.08	0.61	0.32	.75	7.35	0.92	7.65	0.54	1.68	.10
Conc	3.37	1.03	3.42	0.91	0.30	.76	3.96	0.90	3.77	1.02	0.83	.41
SemDen	0.52	0.10	0.53	0.10	0.82	.42	0.51	0.13	0.51	0.12	0.01	.99
SemNeigh	1,162.33	1,957.55	1,806.53	2,376.85	1.62	.11	1,518.67	2,317.74	1,474.53	2,405.86	0.08	.94
SemDiv	1.50	0.29	1.48	0.32	0.25	.80	1.47	0.33	1.56	0.28	1.21	.23
AoA	9.62	2.32	9.81	1.94	0.50	.62	8.30	2.87	7.96	1.76	0.60	.55
Valence	5.00	1.25	5.01	1.41	0.05	.96	5.03	1.27	5.15	1.27	0.39	.70
Arousal	4.28	0.84	4.35	0.87	0.44	.66	4.10	0.78	4.17	1.14	0.33	.74
Dominance	5.08	1.02	4.92	1.06	0.88	.38	5.19	0.86	5.34	0.80	0.79	.43
NPhon	4.98	0.34	7.20	0.90	17.85	.00	4.31	0.47	4.25	0.44	0.52	.60
NLet	6.45	0.77	8.70	0.77	16.06	.00	5.42	0.60	5.39	0.55	0.20	.84
NSyll	2.00	0.00	3.00	0.00	–	–	1.78	0.42	1.72	0.45	0.54	.59
Prev	2.24	0.20	2.28	0.22	0.88	.38	2.17	0.31	2.21	0.28	0.46	.65
PKnown	0.994	0.016	0.992	0.016	0.58	.57	0.991	0.022	0.991	0.026	0.04	.97

Abbreviations are the same as in Table 4

## Supplementary Information

Below is the link to the electronic supplementary material.

Supplementary file1 (PDF 122 KB)

## Data Availability

The raw data and stimuli are available at the Open Science Foundation (10.17605/osf.io/68ye4).

## Footnotes

1 One long word, *butaca*, was not in the database and so the means for the long words are based on seven words.

2 A reviewer noted that randomly generated sublists can have characteristics that differ from those of the larger pool from which they were drawn and queried whether this was the case for these stimuli. The supplementary analyses at the Open Science Framework website (https://doi.org/10.17605/osf.io/68ye4) discusses this and suggests the lists in the small random conditions in Experiment 2 and 3 did not differ from pool as a whole.

3 See Guitard et al. (2023) for details on this particular example and how the results differ between fixed lists for everyone compared with randomly generated lists for everyone.
