# Supplementary material for: Word length vs. lexical factors: Re-examining what causes the word-length effect in serial recognition
Source: Mem Cognit. 2025 Sep 8;54(2):467–82. doi: 10.3758/s13421-025-01762-5 (PMC12956956; doi:10.3758/s13421-025-01762-5)
Supplement: Supplementary file 1 — Supplementary file1 (PDF 122 KB) [file 13421_2025_1762_MOESM1_ESM.pdf]

Supplemental Analyses of Stimulus Sets

In Experiment 2 of Guitard et al. (2025), a set of 60 short (2 syllable) words and 60 long (3 syllable) words were equated on 19 dimensions including frequency, contextual diversity, orthographic and phonological neighborhood size and frequency, concreteness, prevalence, semantic density, semantic neighborhood size, semantic diversity, age of acquisition, valence, arousal, and dominance. The words differed in number of phonemes (*M* 4.95, range 4-5 for short versus 7.2, range 6-9 for long), number of letters (*M* 6.55, range 6-7 for short versus 8.7, range 8-10 for long), and number of syllables (2 versus 3).

One manipulation was set size and one condition was called "small random." In this condition, 10 short and 10 long words were randomly sampled from the larger pool for each subject. On each trial, 5 short or 5 long words were randomly sampled from this sub-pool.

A reviewer noted the possibility that these small sub-pools might have different properties than the larger pool. To assess this possibility, we analyzed whether the sub-pools differed from larger pool. First, we identified the 10 short and 10 long words selected for each subject in the small random condition and calculated the means for those words. For example, Subject 1 had the following 10 short words: *ashtray, athlete, checkup, gospel, nephew, ordeal, outlook, rainbow, runway, termite*. The mean phonological Levenshtein distance (PLD) for these 10 words is 2.730. We did the same for the other 69 subjects and the mean PLD for all subjects is 2.721. We used a two-tailed one-sample *z* test to determine whether the mean of the sub-pools (2.721) differed from the mean of the large pool (2.708, *SD* = 0.399). The z test is appropriate because both the mean and standard deviation of the population (the full pool) are known. We did this for all 21 dimensions we considered and for both short and long sets. The tables below

show the means and *p* values for each dimension for the short words (top table) and long words (bottom table). All *p* values are greater than 0.05, indicating no evidence for a difference.

These results are consistent with the claim that the randomly generated small sub-pools do not differ, over the experiment as a whole, from the large pool.

	Short Words			
	Pool		Sub-pools	*z*
	M	*SD*	*M*	*p*
LgHAL	7.41	1.3	7.35	0.705
LgSubTLWF	2.06	0.45	2.06	0.963
LgSubTLCD	1.90	0.43	1.90	0.986
Ortho_N	0.02	0.13	0.01	0.805
Phono_N	0.00	0.00	0.00	–
OLD	2.73	0.31	2.74	0.776
OLDF	7.06	0.39	7.08	0.647
PLD	2.71	0.40	2.72	0.796
PLDF	7.04	0.72	7.02	0.759
Conc	3.37	1.03	3.36	0.960
SemDen	0.52	0.10	0.51	0.606
SemNeigh	1162.33	1957.55	1209.85	0.839
SemDiv	1.5	0.29	1.51	0.834
AoA	9.62	2.32	9.53	0.771
Valence	5.00	1.25	4.90	0.499
Arousal	4.28	0.84	4.27	0.956
Dominance	5.08	1.02	5.04	0.693
NPhon	4.98	0.34	4.98	0.991
NLet	6.45	0.77	6.48	0.768
NSyll	2.00	0.00	2.00	–
Prev	2.24	0.20	2.24	0.803
PKnown	0.994	0.016	0.99	0.877

	Long Words			
	Pool		Sub-pools	*z*
	M	*SD*	*M*	*p*
LgHAL	7.60	1.46	7.63	0.844
LgSubTLWF	2.12	0.50	2.13	0.903
LgSubTLCD	1.97	0.46	1.98	0.881
Ortho_N	0.00	0.00	0.00	–
Phono_N	0.00	0.00	0.00	–
OLD	2.75	0.15	2.75	0.833
OLDF	7.09	0.41	7.13	0.392
PLD	2.84	0.47	2.83	0.964
PLDF	7.08	0.61	7.09	0.867
Conc	3.42	0.91	3.39	0.795
SemDen	0.53	0.10	0.53	0.883
SemNeigh	1806.53	2376.85	1776.48	0.916
SemDiv	1.48	0.32	1.49	0.867
AoA	9.81	1.94	9.82	0.965
Valence	5.01	1.41	5.06	0.777
Arousal	4.35	0.87	4.37	0.806
Dominance	4.92	1.06	4.92	0.988
NPhon	7.20	0.90	7.17	0.760
NLet	8.70	0.77	8.67	0.720
NSyll	3.00	0.00	3.00	–
Prev	2.28	0.22	2.28	0.866
PKnown	0.99	0.02	0.99	0.771

In Experiment 3 of Guitard et al. (2025) a set of 36 small neighborhood and 36 large neighborhood words was created. These words were equated on dimensions of length (number of phonemes, letters, and syllables) as well as other dimensions. The large neighborhood words had a mean of 7.83 orthographic neighbors compared to 0.22 for the small. They also had a mean of 12.50 phonological neighbors compared to 1.03 for the small. They also differed in OLD (1.48 vs. 2.27) and PLD (1.33 vs. 2.02). As in Experiment 2, set size was manipulated. The same analyses were performed on the sub-pools. The tables below show the means and *p* values for

each dimension for the large neighborhood words (top table) and small neighborhood words (bottom table). All *p* values are greater than 0.05, indicating that the properties of the sub-pools did not differ from the large pool.

	Large Neighborhood Words			
	Pool		Sub-pools	*z*
	M	*SD*	*M*	*p*
LgHAL	7.85	1.42	8.04	0.814
LgSubTLWF	2.36	0.57	2.31	0.971
LgSubTLCD	2.18	0.54	2.17	0.984
Ortho_N	0.22	0.42	7.80	0.902
Phono_N	1.03	1.03	12.44	0.910
OLD	2.27	0.32	1.48	0.770
OLDF	7.27	0.50	7.49	0.747
PLD	2.02	0.26	1.35	0.630
PLDF	7.35	0.92	7.67	0.751
Conc	3.96	0.90	3.79	0.885
SemDen	0.51	0.13	0.51	0.866
SemNeigh	1518.67	2317.74	1382.52	0.749
SemDiv	1.47	0.33	1.56	0.977
AoA	8.30	2.87	8.03	0.742
Valence	5.03	1.27	5.10	0.764
Arousal	4.10	0.78	4.13	0.785
Dominance	5.19	0.86	5.32	0.778
NPhon	4.31	0.47	4.26	0.828
NLet	5.42	0.60	5.41	0.731
NSyll	1.78	0.42	1.73	0.886
Prev	2.17	0.31	2.21	0.853
PKnown	0.991	0.022	0.99	0.806

	Small Neighborhood Words			
	Pool		Sub-pools	*z*
	M	*SD*	*M*	*p*
LgHAL	8.08	1.44	7.79	0.759
LgSubTLWF	2.32	0.60	2.37	0.903
LgSubTLCD	2.17	0.53	2.19	0.878
Ortho_N	7.83	1.96	0.20	0.680
Phono_N	12.50	4.42	1.06	0.784
OLD	1.48	0.14	2.27	0.976
OLDF	7.47	0.46	7.33	0.370
PLD	1.33	0.24	2.02	0.916
PLDF	7.65	0.54	7.36	0.920
Conc	3.77	1.02	3.94	0.866
SemDen	0.51	0.12	0.51	0.949
SemNeigh	1474.53	2405.86	1583.98	0.814
SemDiv	1.56	0.28	1.49	0.695
AoA	7.96	1.76	8.36	0.871
Valence	5.15	1.27	5.00	0.809
Arousal	4.17	1.14	4.07	0.790
Dominance	5.34	0.80	5.20	0.898
NPhon	4.25	0.44	4.29	0.840
NLet	5.39	0.55	5.38	0.597
NSyll	1.72	0.45	1.77	0.877
Prev	2.28	0.22	2.17	0.858
PKnown	0.99	0.02	0.99	0.841

We did not conduct similar analyses on the stimuli from Experiment 1 because the purpose of that experiment was to show that our design replicates previous results when confounded stimuli are used.

Reference

Guitard, D., Neath, I., & Surprenant, A. M. (2025). Word length vs. lexical factors: Re-examining what causes the word length effect in serial recall. Manuscript submitted to *Memory & Cognition.*
